# Cancer-associated fibroblasts drive CXCL13 production in activated T cells via TGF-beta

**DOI:** 10.3389/fimmu.2023.1221532

**Published:** 2023-07-13

**Authors:** Richard A. O’Connor, Begoña Roman Martinez, Lilian Koppensteiner, Layla Mathieson, Ahsan R. Akram

**Affiliations:** ^1^ Centre for Inflammation Research, Institute of Regeneration and Repair, University of Edinburgh, Edinburgh, United Kingdom; ^2^ Cancer Research UK Scotland Centre, Institute of Genetics and Cancer, The University of Edinburgh, Edinburgh, United Kingdom

**Keywords:** cancer associated fibroblast, T cell, CXCL13, Treg, non-small cell lung cancer

## Abstract

**Introduction:**

Tumour-reactive T cells producing the B-cell attractant chemokine CXCL13, in solid tumours, promote development of tertiary lymphoid structures (TLS) and are associated with improved prognosis and responsiveness to checkpoint immunotherapy. Cancer associated fibroblasts are the dominant stromal cell type in non-small cell lung cancer (NSCLC) where they co-localise with T cells and can influence T cell activation and exhaustion. We questioned whether CAF directly promote CXCL13-production during T cell activation.

**Methods:**

We characterised surface markers, cytokine production and transcription factor expression in CXCL13-producing T cells in NSCLC tumours and paired non-cancerous lung samples using flow cytometry. We then assessed the influence of human NSCLC-derived primary CAF lines on T cells from healthy donors and NSCLC patients during activation *in vitro* measuring CXCL13 production and expression of cell-surface markers and transcription factors by flow cytometry.

**Results:**

CAFs significantly increased the production of CXCL13 by both CD4^+^ and CD8^+^ T cells. CAF-induced CXCL13-producing cells lacked expression of CXCR5 and BCL6 and displayed a T peripheral helper cell phenotype. Furthermore, we demonstrate CXCL13 production by T cells is induced by TGF-β and limited by IL-2. CAF provide TGF-β during T cell activation and reduce availability of IL-2 both directly (by reducing the capacity for IL-2 production) and indirectly, by expanding a population of activated Treg. Inhibition of TGF-β signalling prevented both CAF-driven upregulation of CXCL13 and Treg expansion.

**Discussion:**

Promoting CXCL13 production represents a newly described immune-regulatory function of CAF with the potential to shape the immune infiltrate of the tumour microenvironment both by altering the effector-function of tumour infiltrating T-cells and their capacity to attract B cells and promote TLS formation.

## Introduction

The suppressive influence of the tumour microenvironment restrains adaptive immune responses with the potential to destroy cancer cells. Checkpoint-inhibition overcomes elements of this suppression and represents a major advance in the treatment of non-small cell lung cancer (NSCLC). Identifying the cellular players responsive to checkpoint-inhibition can both illustrate pre-requisites for effective anti-tumour immunity and help predict its efficacy. CD8^+^ cytotoxic lymphocytes (CTL) capable of directly killing tumour cells are considered as the prime target of immunotherapy but CD4^+^ T helper cells and B cells can also contribute to revival of anti-tumour immunity following checkpoint inhibition (1–4). The majority of tumour reactive CTL in the tumour microenvironment (TME) show characteristics of T cell exhaustion including high levels of PD-1 alongside multiple co-inhibitory markers including CTLA-4, Tim3, GITR and CD39 (5). Functionally, although their production of IL-2 and IFN-γ is impaired, tumour-reactive T cells in NSCLC produce high levels of CXCL13 (6). Indeed, elevated levels of CXCL13^+^ T cells is highly predictive of responsiveness to PD-1 blockade in NSCLC (6). The strength of this association was confirmed when a recent meta-analysis, of published single-cell data for CXCL13^+^ CD8^+^ T cells, found their presence correlated with improved responsiveness to checkpoint therapy across all cancers tested (7). Furthermore, numbers of tumour reactive CXCL13^+^ CD8^+^ T cells were increased following anti-PD-1 treatment, indicating that expansion of this population is a hallmark of anti-cancer immunity (7–9).

CXCL13 is chemoattractant chemokine which acts via its receptor CXCR5 to promote lymphocyte recruitment to tertiary lymphoid structures (TLS) (10). TLS are ectopically formed aggregates of lymphoid cells displaying organisational characteristics of lymph nodes, including the presence of mature dendritic cells and T cell clusters adjacent to B cell follicles, which form during chronic inflammation, infection and cancer and promote adaptive responses (11). Lung cancer patients with TLS have a better overall prognosis and long term survival than those without (12) and a high density of follicular B cells in TLS improves clinical outcome (13). CXCL13 has a key role in driving TLS formation (10) and CXCL13-producing T cells in NSCLC are located within TLS (6). In ovarian cancer an increased frequency of CD103^+^ CD8^+^ T cells expressing CXCL13 is associated with increased B cell density (14) further suggesting an important role for CXCL13 producing T cells in driving B cell recruitment and TLS formation. Besides CXCL13 producing CD8+ T cells tumour infiltrating CD4^+^ T cells also display an enhanced capacity for CXCL13 production in lung cancer, breast cancer and ovarian cancer (6, 14–16). In breast cancer (where high levels of CXCL13 expression are an independent prognostic factor) CD4^+^ T cells are the dominant intratumoral source of CXCL13 and correlate with increased B cell infiltration and maturation (15). The presence of CD4^+^ CXCL13^+^ T cells early in TLS formation in ovarian cancer also supports a role for these cells in co-ordinating adaptive immune responses and facilitating development of TLS to maximise their efficiency (16). Thus, intratumoral CXCL13^+^ T cells are indicative of existing anti-tumour immunity and predictive of a positive response to immunotherapy which, in addition to direct tumour cell killing, may derive from their potential to facilitate TLS formation and B cell recruitment.

Chronic antigenic stimulation in the tumour microenvironments promotes T cells exhaustion defined by co-expression of multiple co-inhibitory molecules and a hierarchical loss of effector function (17). Exhaustion is now recognised as adaptive state allowing T cells to survival persistent stimulation under direction of the transcription factor Tox (18). Although production of classical effector cytokines is reduced in exhausted T cells they can still often display efficient cytotoxic responses and consistently produce the highest levels of CXCL13 (6, 7). Factors driving CXCL13-production in the TME *in vivo* remain incompletely defined but *in vitro* TCR stimulation in the presence of TGF-β promotes CXCL13 production while IL-2 acts to limit CXCL13 production (14, 19). Two lines of evidence indicate environmental factors in the TME promote CXCL13-production in tumour reactive T cells. Using tetramer staining in a cohort of Melan-A/MART-1 vaccinated melanoma patients allowed phenotypic and functional comparison of tumour reactive T cells in peripheral blood versus tumour. Circulating melan-A/MART-1 reactive T cells had a greater capacity for IFN-γ production while tumour-infiltrating cells produced more CXCL13 and displayed higher expression of co-inhibitory molecules (20). Using a TCR based lineage-tracking approach Liu et al. showed that T cells in peripheral blood with identical TCR-sequences to CXCL13^+^ intratumoral T cells did not express CXCL13 and were not in the exhausted state (21). Indeed CXCL13-expression was rarely detectable in peripheral T cells. These results indicate antigenic stimulation in conditions found within the TME promote exhaustion and CXCL13 production.

Cancer associated fibroblasts are the most abundant stromal cell type in the TME and co-localise with T cells in the stromal areas of the tumour mass (22). As single cell analyses continues to reveal the functional heterogeneity of CAF populations (23–25) there is increasing recognition of their immunomodulatory capacity (26) and impact on responsiveness to immunotherapy (27, 28). Fibroblasts play essential roles in the organisation of lymphocyte populations from the development of lymphoid tissues, to the compartmentalisation of lymph nodes and in lymphoid neogenesis during infection, inflammation and cancer (29, 30). CAF can influence adaptive immunity in many ways, some negative: retaining T cells in the stroma and preventing migration into the tumour islets (31), limiting proliferation, promoting Treg recruitment (32), and even directly killing activated T cells (33). More recent research, however, highlights positive interactions, through direct antigen presentation (34) and in promoting development of TLS (35). CAF stimulate expression of multiple co-inhibitory markers when present during T cell activation (22, 36, 37) including high levels of PD-1 expression and upregulation of CD39, features found in tumour infiltrating CXCL13 producing T cells. We questioned whether interactions with CAF would influence CXCL13-production during T cell activation *in vitro*. CAF produced TGF-β and restrained IL-2 production favouring increased CXCL13 production demonstrating the capacity to contribute to driving T cell exhaustion during stimulation in the TME.

## Materials and methods

### Ethics statement

Healthy volunteer blood was obtained following informed consent and the study was approved by Lothian Regional Ethics Committee (REC) (REC No: 20-HV-069). Cancer/lung tissue and blood samples were obtained following studies which were approved by NHS Lothian REC and facilitated by NHS Lothian SAHSC Bioresource (REC No: 15/ES/0094) and West of Scotland Research Ethics Service (REC No: 21/WS/0094). All participants provided written informed consent prior to enrolment in the studies.

### Samples and NSCLC tissue digestion

NSCLC tissues and adjacent non-cancerous lung samples were collected from patients undergoing surgical resection with curative intent. Tumours >30mm in diameter had areas from within macroscopic tumour and distal non-cancerous lung dissected by the attending pathologist. Fresh samples were processed immediately or stored in media overnight, then minced as finely as possible with scissors in bijoux prior to incubation with 1 mg/ml Collagenase IV (Merck), 1 mg/ml DNase 1 (Merck) in DMEM (Life technologies) for 1 hr at 37°C with agitation. After digestion, samples were passed through 100 µM filters and then 70 µM filters to remove debris and centrifuged at 350 x g for 5 mins at room temperature. Supernatant was removed prior to red cell lysis (Merck) and counting. Typically single cell suspensions were surface stained and analysed directly by flow cytometry. Cells for cryopreservation were stored in CS10 (STEMCELL technologies) and frozen at -70 before transfer to liquid nitrogen storage.

PBMC from NSCLC patients were isolated from EDTA anti-coagulated whole blood samples collected 1 day prior to surgery and isolated using lymphoprep and Sepmate^®^ tubes (both STEMCELL technologies). PBMC from healthy donors were obtained from consented adults in accordance with local regulations. When not used immediately PBMC were cryopreserved in liquid nitrogen in CS10 media. Cryopreserved healthy donor PBMC were also purchased from STEMCELL technologies.

### Generation of CAF lines

Single cell suspensions from NSCLC tumours were incubated overnight in DMEM 100 U/L penicillin/streptomycin, 2 mM L-glutamine and 10% FCS (all Gibco). Non-adherent cells were washed away and remaining cells grown to confluence in media supplemented with 1 X Insulin-Transferrin-Selenium. At passage cells were washed in PBS prior to treatment with 0.5% Trypsin EDTA for three minutes at 37°C to lift cells. At the third passage (when uniform CAF lines were free of non-CD90+ cells) CAF lines were cryopreserved in CS10 media. All CAF lines used were at passage 3 – 6. At passage 3 and beyond CAF lines were typically FAP^+^, CD29^+^ and PDPN^-^ (**Supplementary Figure 1**). Cell lines were regularly tested for the presence of mycoplasma contamination.

### CAF/T cell co-culture conditions

To analysis chemokine production by CAF were plated at 4 x 10^4^/well in 6 well plates (Corning) and allowed establish prior replacing media with or without addition of 25 ng/ml rIFN-γ, 25 ng/ml rTNF-α (both Biolegend), or a combination of rIFN-γ and rTNF-α as indicated. After 48 hrs supernatants were harvested, centrifuged at 350 x g to remove cells and debris, supernatants were then stored at -20°C prior to analysis. For co-culture with T cells, CAF were plated at 2 x 10^4^/well in 48 well plates in complete media (DMEM plus 10% FBS with addition of Pen/Strep and L-Glutamine (All Life technologies)). T cells were purified from PBMC preps using an EasySep Human T cell isolation kit (STEMCELL technologies) according to the manufacturer's instructions. 4 hrs after plating, when CAF had adhered, purified T cells were plated alone or added to CAF containing wells at 5x10^5^/well in complete media with the additions indicated in individual experiments. T cells were stimulated with anti-CD3 (clone) anti-CD28 (clone) antibodies (both at 1 μg/ml) or with anti-CD3/anti-CD28 coated Beads (DYNAL) at a 1:1 ratio with T cells as indicated. The anti-IL-2 antibody (Clone 5334, R and D systems) was used at 2 μg/ml to neutralise IL-2. To inhibit TGF-β, the ALK inhibitor SB431542 was used at 10 µM (TOCRIS).

### Cytokine/chemokine analysis

Supernatants from co-cultures for analysis of chemokine production were harvested after 96 hrs culture, centrifuged at 350 x g to remove cells and debris. Supernatants were then stored at -20°C prior to analysis. A Legendplex^®^ mix and match bead array assay was used to detect production of CXCL13, CCL22 and CX3CL1 according to the manufacturer’s instructions. CXCL13 production was also measured by ELISA (RnD systems). Active TGF-β was measured by ELISA according to the manufacturer's instructions (Biolegend).

### Flow cytometry

Cells were washed in PBS, dead cells were stained with Zombie UV (Biolegend) according to the manufacturers instructions. Fc receptors were blocked with Trustain FcX (Biolegend) prior to staining with the indicated monoclonal antibodies in PBS 2% FCS (see details in **Supplementary Table 1** for full details of antibodies used). Intracellular cytokine staining was performed using BD cytofix/cytoperm buffers according to the manufacturer's instructions. When intracellular cytokine staining was performed alongside intra-nuclear staining, the Foxp3-permeabilization buffer kit (Invitrogen) was used according to the manufacturer's instructions. It has previously been shown that restimulation is not required to detect CXCL13 production in tumour infiltrating lymphocytes *ex-vivo* (15). We confirmed this in a preliminary experiment (**Supplementary Figure 2**). Consequently, staining of CXCL13 in T cells from tumour and non-cancerous lung samples was carried out directly upon freshly isolated or cryopreserved cells without protein transport inhibition or *in vitro* stimulation. In a subset of experiments performed to detect co-expression of intracellular CXCL13 with cytokines which do require activation and stimulation for detection (TNF-α/IFN-γ (**Supplementary Figure 2**)) cells from tumour and non-cancerous lung digests were incubated for 3hrs with 1 X cell activation cocktail (containing Phorbol-12-myristate 13-acetate, ionomycin, brefeldin A and monensin (Invitrogen)) to stimulate cytokine production. All FACS data was collected on a 6 laser LSR (BD) and analysed using Flowjo Software (BD). Gating strategy to CD4+ and CD8+ populations is shown in **Supplementary Figure 3**.

### Statistical analysis

One way ANOVA with Tukey’s multiple comparisons post-test was used for comparison of multiple groups, results of p=<0.05 were considered significant and individual p values are shown for significant comparisons. Paired T tests were used for comparison of two experimental groups. All statistical analysis was performed using Graphpad Prism software.

### Results

#### PD-1^hi^ CD39^+^ CXCL13-producing T cells can be detected *ex-vivo* in NSCLC

**Figure 1:**
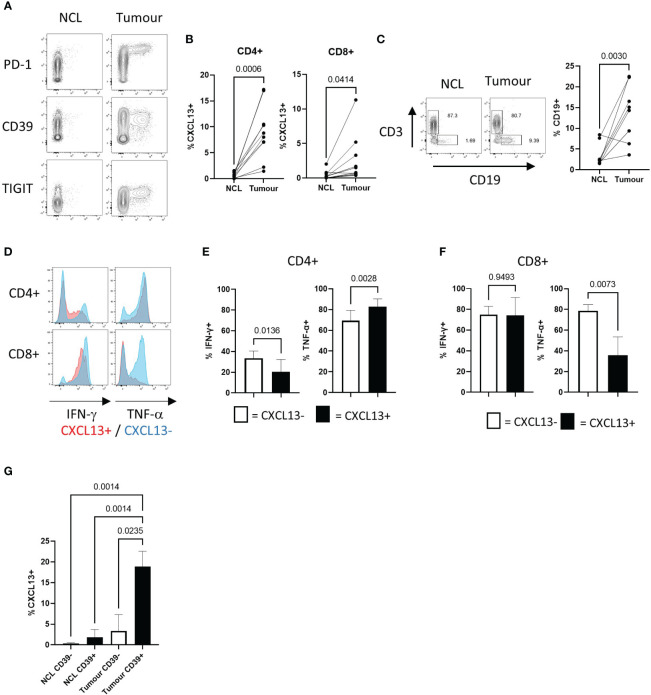
NSCLC Tumours contain recently activated CXCL13 producing T cells expressing multiple co-inhibitory molecules with decreased potential for cytokine production **(A)** Contour plots showing flow cytometry staining for PD-1, CD39, and TIGIT expression against CXCL13 production detected in CD4+ T cells from non-cancerous lung (NCL (left hand panels)) and NSCLC tumour samples ex-vivo (right hand panels). **(B)** Percentage of cells expressing CXCL13 in NCL and Tumour samples (n=10) (within CD4+ or CD8+ populations respectively). Statistical analysis by paired T test. **(C)** Proportions of CD19+ B cells amongst lymphocytes from paired NCL and tumour samples (n=10). Statistical analysis by paired T test. **(D)** Cytokine production in CXCL13- and CXCL13+ Tumour infiltrating T cells (CD4+ in upper panels CD8+ in lower panels). **(E)** IFN-γ production and TNF-α production within CXCL13- and CXCL13+ CD4+ tumour infiltrating T cells (n=5). Statistical analysis by paired T test. **(F)** IFN-γ production and TNF-α production within CXCL13- and CXCL13+ tumour infiltrating CD8+ T cells (n=5). Statistical analysis by paired T test. **(G)**) The frequency of CXCL13+ cells within CD39- and CD39+ populations of CD4+ T cells in NCL and Tumour samples (n=5). Statistical analysis by one way ANOVA with Tukey’s multiple comparison test.

We compared the frequencies of CXCL13^+^ T cells in NSCLC tumours and matched non-cancerous lung samples using flow cytometry. CXCL13-expressing cells showed high levels of PD-1 expression (as previously described (6)) TIGIT and the ectonucleotidase CD39 (6, 7) (**Figure 1A**). The local enrichment of CXCL13-producing cells was found in both CD4^+^ and CD8^+^ populations (**Figure 1B**) and associated with a significantly increased frequency of B cells in tumours compared to non-cancerous lung (**Figure 1C**). Expression of the ectonucleotidase CD39 is associated with terminal exhaustion resulting from chronic antigenic stimulation (38). Consequently, tumour reactive T cells express high levels of CD39 (39, 40) and this can distinguish them from tumour infiltrating bystander-T cells that do not express CD39 (41). CXCL13+ tumour infiltrating T cells also expressed higher levels of CD137, indicative of recent activation (**Supplementary Figure 4A**). Functionally CXCL13^+^ T cells were able to produce IFN-γ and TNF-α (**Figures 1D–F**). Notably however, CXCL13+ CD8+ T cells showed a reduced capacity for TNF-α production compared to their CXCL13- counterparts (**Figures 1D, F**). CXCL13+ T cells displayed a reduced capacity for IL-2 production (**Supplementary Figure 4B**) in agreement with previously reported low levels of IL-2 production in CD39+ tumour infiltrating T cells (42). Increased expression of the exhaustion-associated transcription factor Tox, is characteristic of CD39+ T cells (18) (**Supplementary Figure 4C**) and reduced expression of IL-2 (42) are. Notably however, the frequency of CXCL13^+^ cells was significantly greater in tumour infiltrating CD39^+^ T cells versus those in non-cancerous lung, suggesting factors within the tumour microenvironment promote CXCL13 production (**Figure 1G**).

### CAF increase CXCL13-production during T cell activation

**Figure 2:**
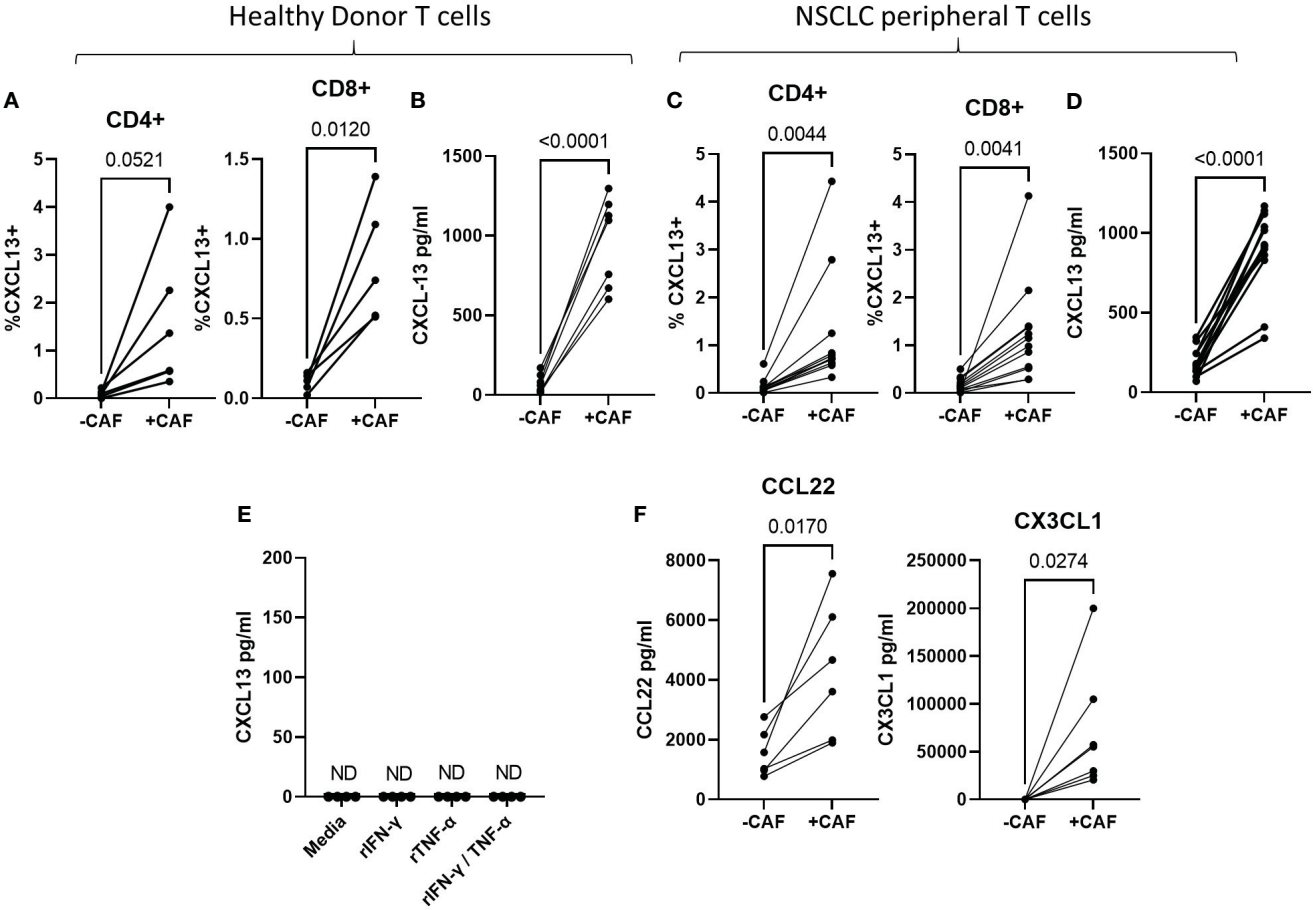
CAF increase CXCL13 production during T cell activation T cells were activated with anti-CD3/anti-CD28 coated beads for 96 hrs in the presence or absence of CAF as indicated. **(A)** Frequency of CXCL13+ cells within CD4+ and CD8+ populations of T cells from healthy donors as determined by flow cytometry in the presence of absence of CAF (n=6 across 4 repeat experiments). **(B)** CXCL13 levels in the supernatant of Healthy donor T cells activated in the presence or absence of CAF (n=7 across three independent experiments). **(C)** Frequencies of CXCL13+ cells within CD4+ and CD8+ T cell populations from NSCLC-patients activated in the presence or absence of CAF (n=12). **(D)** CXCL13 production by T cells from NSCLC patients after 96s hrs activation in the presence, or absence of CAF (n=12). **(E)** Measurement of CXCL13 in the supernatant of 4 CAF lines cultured in the presence of the indicated recombinant cytokines. **(F)** Levels of CCL22 and CX3CL1 produced during activation of healthy donor T cells in the presence or absence of CAF (n=7 across 3 independent experiment). All statistical test performed arepaired T tests.

The majority of tumour-infiltrating T cells are found in stromal areas in close association with CAF (43) which promote expression of CD39 and multiple exhaustion markers during T cell activation *in vitro* (36, 37). To determine whether CAF-promote CXCL13 production during T cell activation we activated peripheral T cells in the presence or absence of primary CAF-lines and measured CXCL13 production by flow cytometry and cytokine bead array. CAF increased the proportion of CD4^+^ and CD8^+^ T cells producing CXCL13 (**Figure 2A**) and the amount of secreted CXCL13 (**Figure 2B**). CAF-induced CXCL13 production by T cells derived from healthy donors (**Figures 2A, B**) and NSCLC patients (**Figures 2C, D**). In the absence of activated T cells CAF alone did not produce measurable levels of CXCL13 (**Figure 2E**). Although we found no evidence for CXCL13 production by CAF in the steady state CAF can produce a number of chemokines able to influence adaptive immune responses (reviewed (44)). As cytokines produced by activated T cells can drive high levels of chemokine production by CAF (and IFN-γ and TNF-α often display synergistic effects in this respect) we tested whether exposure to recombinant cytokines would induce CXCL13 production in CAF. Neither IFN-γ or TNF-α nor a combination of the two induced CXCL13 production in any of the CAF lines tested (**Figure 2E**) leading us to conclude that T cells are the source of CXCL13 in co-cultures. Notably co-culture with CAF also induced increased production of CCL22 and CX3CL1 (**Figure 2F**) which are also associated with TLS formation (45, 46) and drawing T peripheral helper cells to sites of inflammation (47). These results indicate the interactions with CAF during T cell activation increase production of chemokines involved in TLS formation.

### CAF-induced CXCL13 producing T cells show characteristics of T peripheral helper cells

**Figure 3:**
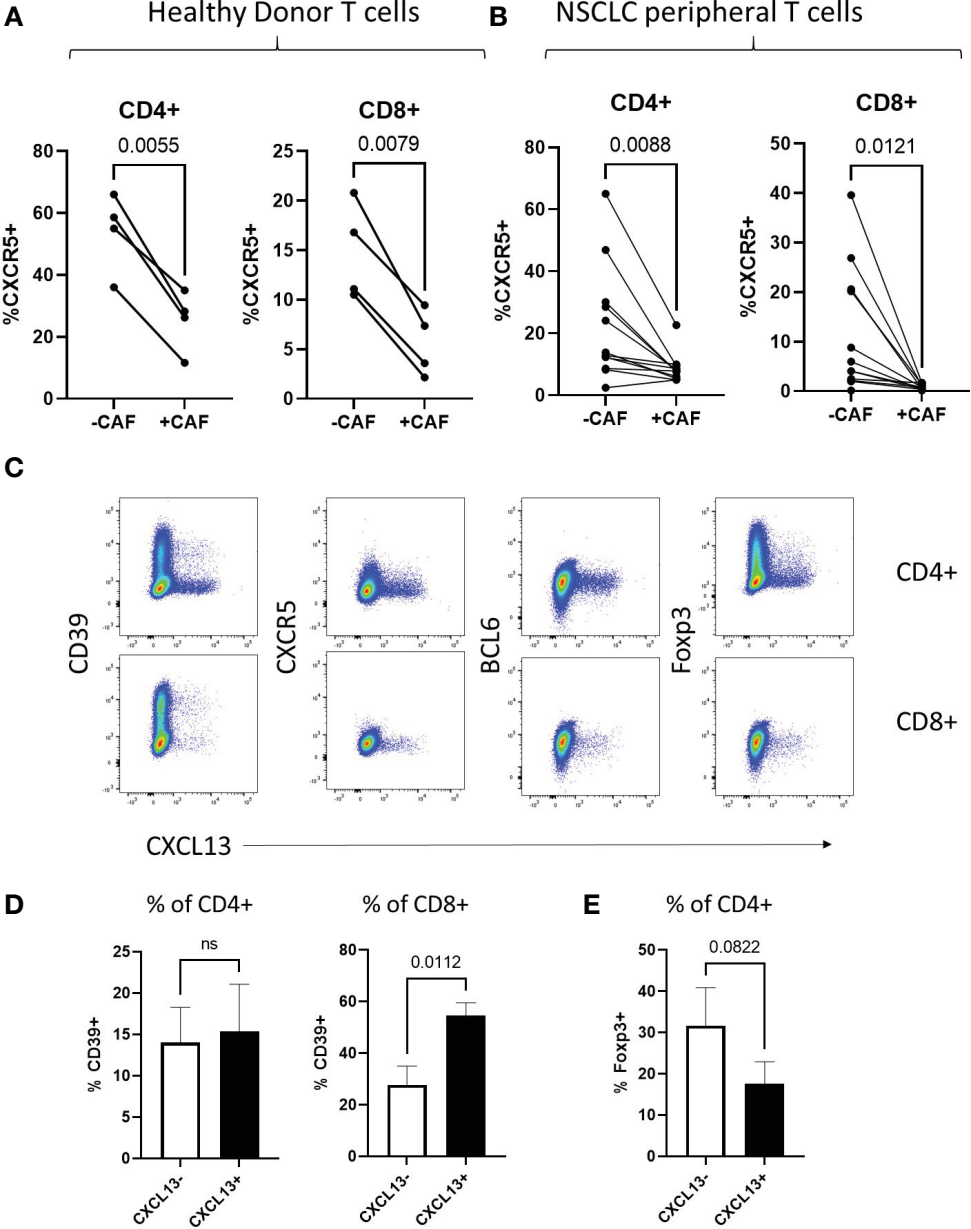
CAF-induced CXCL13 producing T cells show characteristics of T peripheral helper cells **(A)** Expression of CXCR5 within populations of CD4+ and CD8+ peripheral blood T cells from healthy donors activated in the presence and absence of CAF detected by flow cytometry (n=4). **(B)** Expression of CXCR5 within populations of CD4+ and CD8+ peripheral blood T cells from NSCLC patients activated in the presence and absence of CAF detected by flow cytometry (n=12). **(C)** CXCL13 production (X-axis) in relation to expression of CD39, CXCR5, BCL6 and Foxp3 (Y-axis) from CD4+ (upper panels) and CD8+ (lower panels) of T cells from Healthy donors activated in the presence of CAF. **(D)** Expression of CD39 within populations of CXCL13- and CXCL13+ CD4+ and CD8+ T cells after activation in the presence of CAF. **(E)** Expression of Foxp3 within CXCL13- and CXCL13+ populations of CD4+ T cells after activation in the presence of CAF. All statistical test performed are paired T tests.

Classical T follicular helper cells (T_FH_) in secondary lymphoid organs are characterised by expression of high levels of CXCR5 and the transcription factor BCL-6 in contrast to T peripheral helper cells associated with TLS in chronic-inflammation (48, 49) and cancer (15, 50) (reviewed (47)). While CAF-increased production of CXCL13 during T cell activation, we saw a corresponding decrease in expression of the CXCL13-receptor CXCR5 both in T cells from healthy donors and NSCLC patients (**Figures 3A, B** respectively). T cells induced to express CXCL13 during co-culture with CAF lacked expression of both CXCR5 and BCL6 (**Figure 3C**) displaying a T-peripheral helper cell phenotype as reported in nasopharyngeal (50) and breast cancer (15). In contrast to TIL sampled *ex-vivo* in NSCLC (**Figure 1A**) CXCL13^+^ cells generated *in vitro* did not all express CD39, indicating that chronic antigenic stimulation and progression to a terminally exhausted CD39^+^ state is not a prerequisite for CXCL13 production (**Figures 3C, D**). There was however an increased expression of CD39 in CXCL13^+^ versus CXCL13^-^ CD8^+^ T cells (**Figure 3D**). CAF-induced CXCL13^+^ CD4^+^ T cells contained a lower frequency of cells expressing the Treg associated transcription factor Foxp3 than was seen within the CXCL13^-^ population (**Figures 3C, E**). This is in line with previous findings from *in vitro* generation of CXCL13-producing cells using recombinant cytokines (19), RA (51) and breast cancer (15) studies.

### CAF promote expansion of Tregs and limit IL-2 production during T cell activation

**Figure 4:**
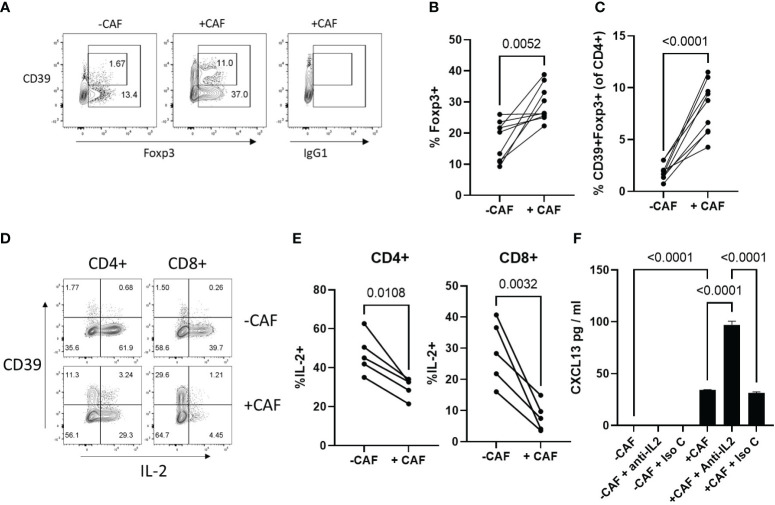
CAF promote expansion of Tregs and limit IL-2 production during T cell activation **(A)** Expression of CD39 (Y-axis) and Foxp3 (X-axis) or isotype matched control for Foxp3 staining (IgG1) within the CD4+ T cell population after activation in the absence (left hand panel) or presence (middle/right panels) of CAF. **(B)** Proportion of CD4+ T cells expressing Foxp3 after 96hrs activation in the presence or absence of CAF (n=9 across 4 independent experiments). Statistical analysis by paired T test. **(C)** Proportion of CD4+ T cells co-expressing CD39 and Foxp3 after 96hrs activation in the presence or absence of CAF (n=9 across 4 independent experiments). Statistical analysis by paired T test. **(D)** Expression of CD39 (Y-axis) and IL-2 (X-axis) in CD4+ and CD8+ T cells activated in the presence (lower panels) or absence (upper panels) of CAF. **(E)** Proportion of CD4+ (left hand panel) and CD8+ T cells (right hand panel) producing IL-2 (n=5 across 2 independent experiments). Statistical analysis by paired T test. **(F)** CXCL13 production by T cells, activated in the presence or absence of CAF, with the indicated addition of either anti-IL2 or isotype matched control antibody (both at 2 μg/ml) data is from one of two independent experiments analysed by ANOVA with Tukey’s multiple comparison test.

Breast cancer derived CAF have been shown to increase the proportion of suppressive Foxp3^+^ Treg (32, 52) which express the high-affinity IL-2 receptor (CD25) and can influence T cell activation and survival by limiting availability of IL-2 (53). As IL-2 acts to limit CXCL13 production during T cell activation, we questioned whether NSCLC-derived CAF promoted expansion of Tregs. There was an increased frequency Foxp3 Treg in the presence of CAF and a greater proportion of these expressed CD39 (**Figures 4A–C**). CAF significantly reduced proportions of IL-2 producing T cells (**Figures 4D, E**). Impaired IL-2 production was most profound in CD8+ T cells and was accompanied by an increased frequency of CD39^+^, T cells which express low levels of IL-2 (40, 54) (**Figures 4D, E**). STAT5 signalling induced by IL-2 can limit CXCL13-prduction during T cell activation (15, 19) suggesting that in the presence of CAF reduced production of IL-2 in combination with increased IL-2-consumption by Treg may be sufficient to drive elevated CXCL13-production. To test this we assessed the impact of an IL-2-neutralising antibody on CXCL13 production. Neutralising IL-2 did not increase CXCL13-production during activation of T cells in the absence of CAF suggesting an additional factor is needed to induce CXCL13-production (**Figure 4F**). In the presence of CAF, however, anti-IL-2 led to a significant increase in CXCL13 production indicating that even the reduced levels of IL-2 produced in co-cultures are sufficient to limit potential CXCL13 production (**Figure 4F**).

### TGF-β drives CAF-induced CXCL13 production

**Figure 5:**
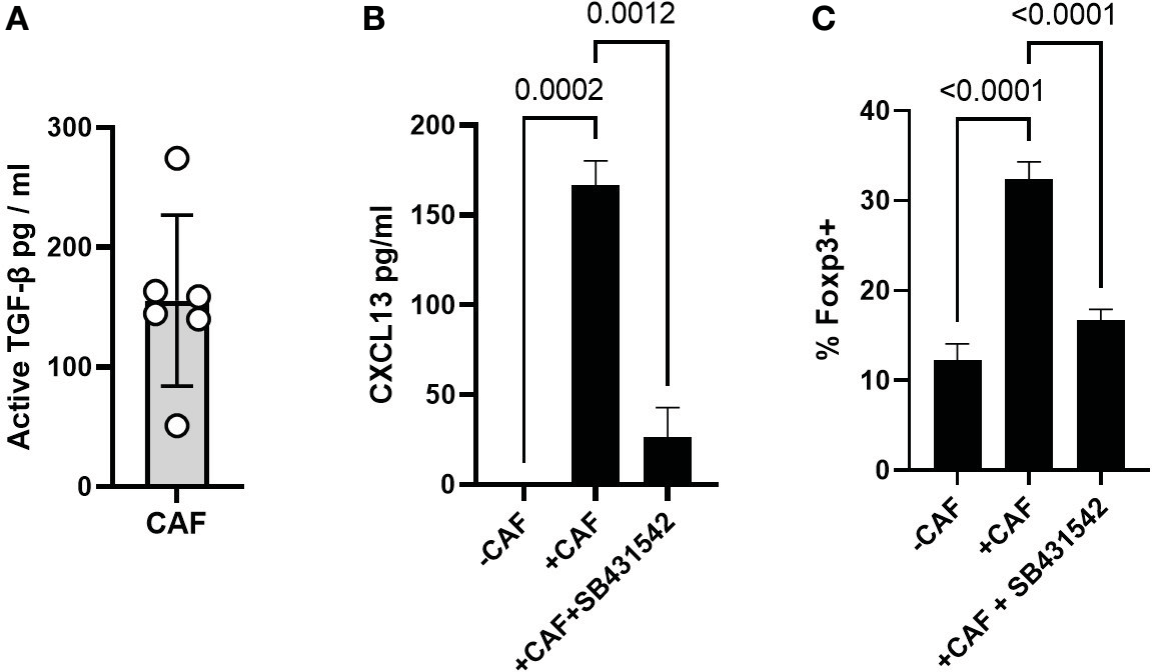
CAF derived TGF-β drives CXCL13 production and Foxp3 expression **(A)** Production of active-TGF-β by CAF *in vitro*, 6 biological replicates from independently derived CAF lines. **(B)** CXCL13 production by T cells activated alone, in the presence of CAF, and in the presence of CAF + SB431542. One representative experiment of 6 independent experiments (7 biological repeats) is shown. **(C)** Expression ofFoxp3 within the CD4+ T cell population after 96 hrs activation alone, in the presence of CAF, and in the presence of CAF plus SB431542 results from one of three independent experiments (5 biological repeats) are shown. Statistical analysis is one way ANOVA with Tukey’s multiple comparison test.

CAF are a known source of TGF-β (55) which is the major driver of CXCL13 in activated T cells (15, 19). We first confirmed that our CAF lines produced active TGF-β *in vitro* (**Figure 5A**). We next assessed the potential role of TGF-β by using the TGF-β-receptor signalling inhibitor SB431542 (56). SB431542 has previously been shown to prevent the phosphorylation of SMAD2 and upregulation of PD-1 in response to TGF-β during T cell activation (57). CAF-induced CXCL13 production was significantly decreased in the absence of TGF-β signalling (**Figure 5B**). Induction of Foxp3-expression *in-vitro* is also dependent upon TGF-β and SB431542 blocked the CAF-induced expansion of Foxp3+ Tregs (**Figure 5C**). Taken together these results demonstrate that TGF-β produced by CAF induces expression of CXCL13 in activated T cells and increases the frequency of Foxp3+ Treg thus mimicking during *in vitro* activation functional characteristics of T cells recovered from the Tumour microenvironment.

## Discussion

Increased understanding of the crosstalk between CAF and T cells in the TME has extended the scope of these interactions beyond their widely reported negative impact (reviewed (58)). Recent reports have focused on the importance of CAF as antigen presenting cells (34) and highlighted their role in driving TLS formation (35), illustrating beneficial roles for CAF promoting factors associated with improved prognosis. Several studies have shown CXCL13^+^ T cells are tumour reactive (59–61) and co-expression of CD39 and CXCL13 allows effective enrichment of tumour reactive CD4+ T cells in NSCLC (59). Functionally CXCL13 drives formation of TLS (10) which are associated with both better prognosis (4, 13) and improved responsiveness to immunotherapy (62). The presence of tumour-infiltrating CXCL13^+^ T cells is the best predictor of responsiveness to anti-PD-1/anti-PDL1 efficacy (6, 7, 63). Experimentally, the relationship between CXCL13 and checkpoint inhibition was established in a recent study showing that peri-tumoral administration of recombinant CXCL13 synergises with anti-PD-1 treatment to decrease tumour growth (64). This illustrates the importance of CXCL13-producing T cells as an indicator of pre-existing anti-tumour immunity with the potential for amplification during successful immunotherapy. Our demonstration that CAF directly promote gain of CXCL13-production shows their potential to influence T cell function, favouring co-ordination and expansion of the adaptive immune response.

CXCL13^+^ TIL in NSCLC predominantly display a terminally exhausted (Tex) PD-1^hi^ phenotype (6, 7) and in keeping with previous observations the CXCL13+ cells we identified *ex-vivo* were uniformly CD39^+^. However, although exhaustion prevails in tumour infiltrating T cells, circulating populations of tumour reactive T cells exist which are not yet terminally exhausted. In melanoma patients, for example, intratumoral-tumor reactive T cells showed increased expression of exhaustion markers, reduced IFN-γ production and increased CXCL13 production compared to circulating tumour reactive T cells (as identified by tetramer staining) (20). Similarly in lung cancer the phenotypic and functional profiles of Tumour reactive T cells showed signs of exhaustion and CXCL13 production exclusively in Tumour infiltrating T cells and not in circulating peripheral T cells with shared specificity (7–9). These data suggest re-encounter with antigen in the suppressive tumour microenvironment promotes exhaustion locally and TGF-β production by CAF-could be a factor contributing up upregulation of exhaustion markers and CXCL13 production. Notably, in both NSCLC and Melanoma, there is an increase in the proportion of CXCL13^+^ T cells in the TME following anti-PD-1-treatment, which lack markers of terminal exhaustion displaying a phenotype of T-exhausted precursor cells (Texp) (21). Terminal exhaustion is a relatively stable state (18, 65) and temporal and RNA-velocity analysis describe a one-way progression from Texp to Tex (21). This suggests that the increased frequency of CXCL13^+^Texp in NSCLC post anti-PD-1 results from decreased pressure within the TME on newly infiltrating tumour reactive T cells to progress towards terminal differentiation upon stimulation, rather than phenotypic change in existing exhausted T cells. If the signals promoting expansion of Texp versus Tex were better characterised this could be exploited in cellular therapies aimed at enriching for T cells with greater proliferative potential capable of promoting TLS formation and strengthening co-ordinated adaptive immunity.

CAF are heterogeneous *in vivo* and phenotypically distinct subsets have unique characteristics (reviewed (30)). In breast cancer for example a subset of Fibroblast Activation Protein (FAP)^+^ CD29^+^ CAF designated CAF-S1, have an enhanced capacity to recruit and expand Treg (32). We found that co-culture with NSCLC-derived CAF significantly increased the proportion of Foxp3^+^ Tregs and the frequency of CD39^+^ Treg. Highly activated CD39^+^ Tregs show enhanced stability and suppressive function under inflammatory conditions due to decreased expression of the IL-6R (66). NSCLC-derived CAF expanded a population of Treg phenotypically similar to those found in the tumour microenvironment demonstrating they influence both conventional and regulatory T cell responses.

Whether the influence of CAF on local immunity changes during the development of a tumour, and how this reflects changes in the dominant CAF subtype, has not yet been finely dissected in human studies. Elegant work in murine models however, has clearly shown that the CAF population is dynamic and that its composition changes during tumour maturation from an early dominance of immune–interacting CAF progressing through to desmoplastic and more contractile forms (67). Thus the outcome of CAF-T cell interactions *in vivo* will be impacted by the representation of distinct CAF subsets present in the tumour. We used a standard protocol for the generation of CAF (68) and the resultant CAF lines were uniformly FAP+CD29+ and typically PDPN-. Removal from the complexity of the tumour microenvironment and the resultant loss of niche pressures coupled with maintenance in 2D culture limits the capacity of this relatively homogenous population to reflect CAF-heterogeneity seen *in vivo* (25). Whether the distinct subsets of NSCLC CAF identified by single cell sequencing (23, 69) have differential capacities to promote CXCL13-production, or expansion of Foxp3^+^ Treg is a pertinent question for future studies.

A subset of FAP^+^, podoplanin^+^ fibroblasts termed “Immunomodulatory fibroblasts” are critical regulators of TLS formation during inflammation (70). Experimental depletion of FAP^+^ cells decreased TLS formation during viral infection directly demonstrating their importance. Interactions with T cells ignite the potential to drive TLS formation via ICOS/ICOS-L interactions which promote LTa3 and CXCL13 production (71). Fibroblasts also drive TLS-formation in a melanoma model wherein they develop characteristics of lymphoid tissue organiser cells in response to TNF-receptor signalling (35). The potential to harness this capacity is illustrated by experimental manipulation of the stromal cell compartmentment, in mouse models, which can directly promote TLS formation. Subcutaneous implantation of a CXCL13-producing fibroblastic reticular cell line, of lymph node origin, induced functional TLS formation, increased immune cell recruitment and enhanced anti-tumour immunity (72). We found no evidence of NSCLC-CAF producing CXCL13 in the steady state *in vitro* nor in response to cytokines produced by activated T cells. However, this does not rule out the prossibilty that CAF may contribute to TLS formation *in vivo* and the comparative analysis of chemokine expression in CAF subsets at distinct stages of disease progression could help reveal their potential contribution.

In his elegant second touch hypothesis Klaus Ley postulated that complete polarization and full gain of effector function is only achieved consequent to a “second touch” provided by encounter with cognate antigen in the tissue setting (73). This theory fits well with gain of CXCL13-producing capacity being restricted to tumour-infiltrating T cells and absent in circulating tumour reactive T cells (7). Interaction with antigen presenting cells in the TME promotes CXCL13 production as demonstrated by sequencing of physically interacting cells in human NSCLC (74) confirming the requirement for TCR stimulation *in situ* to induce CXCL13 production, previously described *in vitro* (14, 19). Immune-interacting fibroblast subtypes, with antigen presenting capacity, have been described in cancer (Reviewed (26)). CAF can process and present antigen to CD8+ T cells (33) and the recent demonstration of MHC-II-restricted antigen presentation to CD4^+^ T cells, *in situ* in NSCLC, provides the first direct evidence that fibroblasts productively interact with CD4^+^ T cells in solid tumours (34). Thus, the potential for CAF to interact with Tumour reactive T cells in the TME and influence their phenotype and function is established.

During chronic inflammation the immune response, unable to eradicate the target antigen, must be tempered to avoid immunopathology. Environmental feedback, via increased expression and engagement of co-inhibitory receptors, promotes an alteration in strategy, switching from proliferation and production of inflammatory cytokines towards exhaustion and increased local accumulation of Treg. Retaining cytotoxicity while increasing production of chemokines such as CXCL13, to increase TLS formation and strengthen humoral as well as cell mediated immunity, could prevent dissemination of an infectious agent, or cancer, which can be contained but not eliminated. In this sense, it seems reasonable that the “second touch” received during antigen encounter in chronically inflamed tissue, should convey the need to promote immune-co-ordination and regulation. Stromal cells, activated during chronic inflammation, are well placed to convey this message. In line with this potential, our results identify three ways in which CAF may modulate the local immune responses: by restricting IL-2 production in activated T cells, by promoting expansion of Treg (which can indirectly limit the availability of IL-2) and by directly driving CXCL13-production via TGF-β. Whether these traits are associated with distinct subsets of CAF, develop at distinct stages in tumour progression, influence responsiveness to immunotherapy or can be manipulated therapeutically are important questions for future research.

## Data availability statement

The raw data supporting the conclusions of this article will be made available by the authors, without undue reservation.

## Ethics statement

The studies involving human participants were reviewed and approved by Lothian Regional Ethics Committee/NHS Lothian. The patients/participants provided their written informed consent to participate in this study.

## Author contributions

RO’C and AA contributed to the conception and design of the study. RO’C, BM, LK and LM performed the experiments, collected the data and analysed the results. RO’C wrote the first draft of the manuscript. All authors contributed to manuscript revision, read, and approved the submitted version.

## References

[1] Tay RE Richardson EK Toh HC. Revisiting the role of CD4(+) T cells in cancer immunotherapy-new insights into old paradigms. Cancer Gene Ther (2021) 28(1-2):5–17. doi: 10.1038/s41417-020-0183-x 32457487 PMC7886651

[2] Helmink BA Reddy SM Gao J Zhang S Basar R Thakur R. B cells and tertiary lymphoid structures promote immunotherapy response. Nature. (2020) 577(7791):549–55. doi: 10.1038/s41586-019-1922-8 PMC8762581 31942075

[3] Petitprez F de Reynies A Keung EZ Chen TW Sun CM Calderaro J. B cells are associated with survival and immunotherapy response in sarcoma. Nature. (2020) 577(7791):556–60. doi: 10.1038/s41586-019-1906-8 31942077

[4] Cabrita R Lauss M Sanna A Donia M Skaarup Larsen M Mitra S. Tertiary lymphoid structures improve immunotherapy and survival in melanoma. Nature. (2020) 577(7791):561–5. doi: 10.1038/s41586-019-1914-8 31942071

[5] Thommen DS Schreiner J Muller P Herzig P Roller A Belousov A. Progression of lung cancer is associated with increased dysfunction of T cells defined by coexpression of multiple inhibitory receptors. Cancer Immunol Res (2015) 3(12):1344–55. doi: 10.1158/2326-6066.CIR-15-0097 26253731

[6] Thommen DS Koelzer VH Herzig P Roller A Trefny M Dimeloe S. A transcriptionally and functionally distinct PD-1(+) CD8(+) T cell pool with predictive potential in non-small-cell lung cancer treated with PD-1 blockade. Nat Med (2018) 24(7):994–1004. doi: 10.1038/s41591-018-0057-z 29892065 PMC6110381

[7] Liu B Zhang Y Wang D Hu X Zhang Z. Single-cell meta-analyses reveal responses of tumor-reactive CXCL13(+) T cells to immune-checkpoint blockade. Nat Cancer (2022) 3(9):1123–36. doi: 10.1038/s43018-022-00433-7 36138134

[8] Caushi JX Zhang J Ji Z Vaghasia A Zhang B Hsiue EH. Transcriptional programs of neoantigen-specific TIL in anti-PD-1-treated lung cancers. Nature. (2021) 596(7870):126–32. doi: 10.1038/s41586-021-03752-4 PMC8338555 34290408

[9] Eberhardt CS Kissick HT Patel MR Cardenas MA Prokhnevska N Obeng RC. Functional HPV-specific PD-1(+) stem-like CD8 T cells in head and neck cancer. Nature. (2021) 597(7875):279–84. doi: 10.1038/s41586-021-03862-z PMC10201342 34471285

[10] Luther SA Lopez T Bai W Hanahan D Cyster JG. BLC expression in pancreatic islets causes b cell recruitment and lymphotoxin-dependent lymphoid neogenesis. Immunity. (2000) 12(5):471–81. doi: 10.1016/S1074-7613(00)80199-5 10843380

[11] Dieu-Nosjean MC Goc J Giraldo NA Sautes-Fridman C Fridman WH. Tertiary lymphoid structures in cancer and beyond. Trends Immunol (2014) 35(11):571–80. doi: 10.1016/j.it.2014.09.006 25443495

[12] Dieu-Nosjean MC Antoine M Danel C Heudes D Wislez M Poulot V. Long-term survival for patients with non-small-cell lung cancer with intratumoral lymphoid structures. J Clin Oncol (2008) 26(27):4410–7. doi: 10.1200/JCO.2007.15.0284 18802153

[13] Germain C Gnjatic S Tamzalit F Knockaert S Remark R Goc J. Presence of b cells in tertiary lymphoid structures is associated with a protective immunity in patients with lung cancer. Am J Respir Crit Care Med (2014) 189(7):832–44. doi: 10.1164/rccm.201309-1611OC 24484236

[14] Workel HH Lubbers JM Arnold R Prins TM van der Vlies P de Lange K. A transcriptionally distinct CXCL13(+)CD103(+)CD8(+) T-cell population is associated with b-cell recruitment and neoantigen load in human cancer. Cancer Immunol Res (2019) 7(5):784–96. doi: 10.1158/2326-6066.CIR-18-0517 30872264

[15] Gu-Trantien C Migliori E Buisseret L de Wind A Brohee S Garaud S. CXCL13-producing TFH cells link immune suppression and adaptive memory in human breast cancer. JCI Insight (2017) 2(11):e91487. doi: 10.1172/jci.insight.91487 28570278 PMC5453706

[16] Ukita M Hamanishi J Yoshitomi H Yamanoi K Takamatsu S Ueda A. CXCL13-producing CD4+ T cells accumulate in the early phase of tertiary lymphoid structures in ovarian cancer. JCI Insight (2022) 7(12):e157215. doi: 10.1172/jci.insight.157215 35552285 PMC9309049

[17] Wherry EJ Kurachi M. Molecular and cellular insights into T cell exhaustion. Nat Rev Immunol (2015) 15(8):486–99. doi: 10.1038/nri3862 PMC4889009 26205583

[18] Scott AC Dundar F Zumbo P Chandran SS Klebanoff CA Shakiba M. TOX is a critical regulator of tumour-specific T cell differentiation. Nature. (2019) 571(7764):270–4. doi: 10.1038/s41586-019-1324-y PMC7698992 31207604

[19] Kobayashi S Watanabe T Suzuki R Furu M Ito H Ito J. TGF-beta induces the differentiation of human CXCL13-producing CD4(+) T cells. Eur J Immunol (2016) 46(2):360–71. doi: 10.1002/eji.201546043 PMC5063156 26541894

[20] Baitsch L Baumgaertner P Devevre E Raghav SK Legat A Barba L. Exhaustion of tumor-specific CD8(+) T cells in metastases from melanoma patients. J Clin Invest (2011) 121(6):2350–60. doi: 10.1172/JCI46102 PMC3104769 21555851

[21] Liu B Hu X Feng K Gao R Xue Z Zhang S. Temporal single-cell tracing reveals clonal revival and expansion of precursor exhausted T cells during anti-PD-1 therapy in lung cancer. Nat Cancer (2022) 3(1):108–21. doi: 10.1038/s43018-021-00292-8 35121991

[22] Koppensteiner L Mathieson L O'Connor RA Akram AR. Cancer associated fibroblasts - an impediment to effective anti-cancer T cell immunity. Front Immunol (2022) 13:887380. doi: 10.3389/fimmu.2022.887380 35479076 PMC9035846

[23] Lambrechts D Wauters E Boeckx B Aibar S Nittner D Burton O. Phenotype molding of stromal cells in the lung tumor microenvironment. Nat Med (2018) 24(8):1277–89. doi: 10.1038/s41591-018-0096-5 29988129

[24] Luo H Xia X Huang LB An H Cao M Kim GD. Pan-cancer single-cell analysis reveals the heterogeneity and plasticity of cancer-associated fibroblasts in the tumor microenvironment. Nat Commun (2022) 13(1):6619. doi: 10.1038/s41467-022-34395-2 36333338 PMC9636408

[25] Mathieson L Koppensteiner L Pattle S Dorward DA O’Connor R Akram A. Subpopulations of cancer-associated fibroblasts expressing fibroblast activation protein and podoplanin in non-small cell lung cancer are a predictor of poor clinical outcome. bioRxiv (2022). doi: 10.1101/2022.09.28.509919 PMC11130154 38582812

[26] Tsoumakidou M. The advent of immune stimulating CAFs in cancer. Nat Rev Cancer (2023) 23:258–69. doi: 10.1038/s41568-023-00549-7 36807417

[27] Kieffer Y Hocine HR Gentric G Pelon F Bernard C Bourachot B. Single-cell analysis reveals fibroblast clusters linked to immunotherapy resistance in cancer. Cancer Discovery (2020) 10(9):1330–51. doi: 10.1158/2159-8290.CD-19-1384 32434947

[28] Hu H Piotrowska Z Hare PJ Chen H Mulvey HE Mayfield A. Three subtypes of lung cancer fibroblasts define distinct therapeutic paradigms. Cancer Cell (2021) 39(11):1531–47 e10. doi: 10.1016/j.ccell.2021.09.003 34624218 PMC8578451

[29] Buechler MB Turley SJ. A short field guide to fibroblast function in immunity. Semin Immunol (2018) 35:48–58. doi: 10.1016/j.smim.2017.11.001 29198601

[30] Davidson S Coles M Thomas T Kollias G Ludewig B Turley S. Fibroblasts as immune regulators in infection, inflammation and cancer. Nat Rev Immunol (2021) 21(11):704–17. doi: 10.1038/s41577-021-00540-z 33911232

[31] Feig C Jones JO Kraman M Wells RJ Deonarine A Chan DS. Targeting CXCL12 from FAP-expressing carcinoma-associated fibroblasts synergizes with anti-PD-L1 immunotherapy in pancreatic cancer. Proc Natl Acad Sci U S A (2013) 110(50):20212–7. doi: 10.1073/pnas.1320318110 PMC3864274 24277834

[32] Costa A Kieffer Y Scholer-Dahirel A Pelon F Bourachot B Cardon M. Fibroblast heterogeneity and immunosuppressive environment in human breast cancer. Cancer Cell (2018) 33(3):463–79 e10. doi: 10.1016/j.ccell.2018.01.011 29455927

[33] Lakins MA Ghorani E Munir H Martins CP Shields JD. Cancer-associated fibroblasts induce antigen-specific deletion of CD8 (+) T cells to protect tumour cells. Nat Commun (2018) 9(1):948. doi: 10.1038/s41467-018-03347-0 29507342 PMC5838096

[34] Kerdidani D Aerakis E Verrou KM Angelidis I Douka K Maniou MA. Lung tumor MHCII immunity depends on in situ antigen presentation by fibroblasts. J Exp Med (2022) 219(2):e20210815. doi: 10.1084/jem.20210815 35029648 PMC8764966

[35] Rodriguez AB Peske JD Woods AN Leick KM Mauldin IS Meneveau MO. Immune mechanisms orchestrate tertiary lymphoid structures in tumors via cancer-associated fibroblasts. Cell Rep (2021) 36(3):109422. doi: 10.1016/j.celrep.2021.109422 34289373 PMC8362934

[36] Gorchs L Fernandez Moro C Bankhead P Kern KP Sadeak I Meng Q. Human pancreatic carcinoma-associated fibroblasts promote expression of Co-inhibitory markers on CD4(+) and CD8(+) T-cells. Front Immunol (2019) 10:847. doi: 10.3389/fimmu.2019.00847 31068935 PMC6491453

[37] O’Connor RA Chauhan V Mathieson L Titmarsh H Koppensteiner L Young I. T Cells drive negative feedback mechanisms in cancer associated fibroblasts, promoting expression of co-inhibitory ligands, CD73 and IL-27 in non-small cell lung cancer. OncoImmunology (2021) 10(1):1940675. doi: 10.1080/2162402X.2021.1940675 34290905 PMC8274440

[38] Gupta PK Godec J Wolski D Adland E Yates K Pauken KE. CD39 expression identifies terminally exhausted CD8+ T cells. PloS Pathog (2015) 11(10):e1005177. doi: 10.1371/journal.ppat.1005177 26485519 PMC4618999

[39] Duhen T Duhen R Montler R Moses J Moudgil T de Miranda NF. Co-Expression of CD39 and CD103 identifies tumor-reactive CD8 T cells in human solid tumors. Nat Commun (2018) 9(1):2724. doi: 10.1038/s41467-018-05072-0 30006565 PMC6045647

[40] Canale FP Ramello MC Nunez N Araujo Furlan CL Bossio SN Gorosito Serran M. CD39 expression defines cell exhaustion in tumor-infiltrating CD8(+) T cells. Cancer Res (2018) 78(1):115–28. doi: 10.1158/0008-5472.CAN-16-2684 29066514

[41] Simoni Y Becht E Fehlings M Loh CY Koo SL Teng KWW. Bystander CD8(+) T cells are abundant and phenotypically distinct in human tumour infiltrates. Nature. (2018) 557(7706):575–9. doi: 10.1038/s41586-018-0130-2 29769722

[42] Canale FP Ramello MC Nunez N Bossio SN Piaggio E Gruppi A. CD39 expression defines cell exhaustion in tumor-infiltrating CD8(+) T cells-response. Cancer Res (2018) 78(17):5175. doi: 10.1158/0008-5472.CAN-18-0950 30115702

[43] Duraiswamy J Turrini R Minasyan A Barras D Crespo I Grimm AJ. Myeloid antigen-presenting cell niches sustain antitumor T cells and license PD-1 blockade via CD28 costimulation. Cancer Cell (2021) 39(12):1623–42 e20. doi: 10.1016/j.ccell.2021.10.008 34739845 PMC8861565

[44] Barrett RL Pure E. Cancer-associated fibroblasts and their influence on tumor immunity and immunotherapy. Elife. (2020) 9:e57243. doi: 10.7554/eLife.57243 33370234 PMC7769568

[45] de Chaisemartin L Goc J Damotte D Validire P Magdeleinat P Alifano M. Characterization of chemokines and adhesion molecules associated with T cell presence in tertiary lymphoid structures in human lung cancer. Cancer Res (2011) 71(20):6391–9. doi: 10.1158/0008-5472.CAN-11-0952 21900403

[46] Astorri E Scrivo R Bombardieri M Picarelli G Pecorella I Porzia A. CX3CL1 and CX3CR1 expression in tertiary lymphoid structures in salivary gland infiltrates: fractalkine contribution to lymphoid neogenesis in sjogren's syndrome. Rheumatol (Oxford) (2014) 53(4):611–20. doi: 10.1093/rheumatology/ket401 24324211

[47] Rao DA. T Cells that help b cells in chronically inflamed tissues. Front Immunol (2018) 9:1924. doi: 10.3389/fimmu.2018.01924 30190721 PMC6115497

[48] Manzo A Vitolo B Humby F Caporali R Jarrossay D Dell'accio F. Mature antigen-experienced T helper cells synthesize and secrete the b cell chemoattractant CXCL13 in the inflammatory environment of the rheumatoid joint. Arthritis Rheumatol (2008) 58(11):3377–87. doi: 10.1002/art.23966 18975336

[49] Rao DA Gurish MF Marshall JL Slowikowski K Fonseka CY Liu Y. Pathologically expanded peripheral T helper cell subset drives b cells in rheumatoid arthritis. Nature. (2017) 542(7639):110–4. doi: 10.1038/nature20810 PMC5349321 28150777

[50] Li JP Wu CY Chen MY Liu SX Yan SM Kang YF. PD-1(+)CXCR5(-)CD4(+) Th-CXCL13 cell subset drives b cells into tertiary lymphoid structures of nasopharyngeal carcinoma. J Immunother Cancer (2021) 9(7):e002101. doi: 10.1136/jitc-2020-002101 34253636 PMC8276302

[51] Kobayashi S Murata K Shibuya H Morita M Ishikawa M Furu M. A distinct human CD4+ T cell subset that secretes CXCL13 in rheumatoid synovium. Arthritis Rheumatol (2013) 65(12):3063–72. doi: 10.1002/art.38173 24022618

[52] Givel AM Kieffer Y Scholer-Dahirel A Sirven P Cardon M Pelon F. miR200-regulated CXCL12beta promotes fibroblast heterogeneity and immunosuppression in ovarian cancers. Nat Commun (2018) 9(1):1056. doi: 10.1038/s41467-018-03348-z 29535360 PMC5849633

[53] Vignali DA. Mechanisms of t(reg) suppression: still a long way to go. Front Immunol (2012) 3:191. doi: 10.3389/fimmu.2012.00191 22783262 PMC3389608

[54] Fang F Yu M Cavanagh MM Hutter Saunders J Qi Q Ye Z. Expression of CD39 on activated T cells impairs their survival in older individuals. Cell Rep (2016) 14(5):1218–31. doi: 10.1016/j.celrep.2016.01.002 PMC4851554 26832412

[55] Kalluri R. The biology and function of fibroblasts in cancer. Nat Rev Cancer (2016) 16(9):582–98. doi: 10.1038/nrc.2016.73 27550820

[56] Inman GJ Nicolas FJ Callahan JF Harling JD Gaster LM Reith AD. SB-431542 is a potent and specific inhibitor of transforming growth factor-beta superfamily type I activin receptor-like kinase (ALK) receptors ALK4, ALK5, and ALK7. Mol Pharmacol (2002) 62(1):65–74. doi: 10.1124/mol.62.1.65 12065756

[57] Park BV Freeman ZT Ghasemzadeh A Chattergoon MA Rutebemberwa A Steigner J. TGFbeta1-mediated SMAD3 enhances PD-1 expression on antigen-specific T cells in cancer. Cancer Discovery (2016) 6(12):1366–81. doi: 10.1158/2159-8290.CD-15-1347 PMC5295786 27683557

[58] Baker AT Abuwarwar MH Poly L Wilkins S Fletcher AL. Cancer-associated fibroblasts and T cells: from mechanisms to outcomes. J Immunol (2021) 206(2):310–20. doi: 10.4049/jimmunol.2001203 33397745

[59] Hanada KI Zhao C Gil-Hoyos R Gartner JJ Chow-Parmer C Lowery FJ. A phenotypic signature that identifies neoantigen-reactive T cells in fresh human lung cancers. Cancer Cell (2022) 40(5):479–93 e6. doi: 10.1016/j.ccell.2022.03.012 35452604 PMC9196205

[60] Lowery FJ Krishna S Yossef R Parikh NB Chatani PD Zacharakis N. Molecular signatures of antitumor neoantigen-reactive T cells from metastatic human cancers. Science. (2022) 375(6583):877–84. doi: 10.1126/science.abl5447 PMC8996692 35113651

[61] Zheng C Fass JN Shih YP Gunderson AJ Sanjuan Silva N Huang H. Transcriptomic profiles of neoantigen-reactive T cells in human gastrointestinal cancers. Cancer Cell (2022) 40(4):410–23 e7. doi: 10.1016/j.ccell.2022.03.005 35413272

[62] Lin Z Huang L Li S Gu J Cui X Zhou Y. Pan-cancer analysis of genomic properties and clinical outcome associated with tumor tertiary lymphoid structure. Sci Rep (2020) 10(1):21530. doi: 10.1038/s41598-020-78560-3 33299035 PMC7725838

[63] Litchfield K Reading JL Puttick C Thakkar K Abbosh C Bentham R. Meta-analysis of tumor- and T cell-intrinsic mechanisms of sensitization to checkpoint inhibition. Cell. (2021) 184(3):596–614 e14. doi: 10.1016/j.cell.2021.01.002 33508232 PMC7933824

[64] Sorin M Karimi E Rezanejad M Yu MW Desharnais L McDowell SAC. Single-cell spatial landscape of immunotherapy response reveals mechanisms of CXCL13 enhanced antitumor immunity. J Immunother Cancer (2023) 11(2):e005545. doi: 10.1136/jitc-2022-005545 36725085 PMC9896310

[65] Pauken KE Sammons MA Odorizzi PM Manne S Godec J Khan O. Epigenetic stability of exhausted T cells limits durability of reinvigoration by PD-1 blockade. Science. (2016) 354(6316):1160–5. doi: 10.1126/science.aaf2807 PMC5484795 27789795

[66] Gu J Ni X Pan X Lu H Lu Y Zhao J. Human CD39(hi) regulatory T cells present stronger stability and function under inflammatory conditions. Cell Mol Immunol (2017) 14(6):521–8. doi: 10.1038/cmi.2016.30 PMC5518817 27374793

[67] Davidson S Efremova M Riedel A Mahata B Pramanik J Huuhtanen J. Single-cell RNA sequencing reveals a dynamic stromal niche that supports tumor growth. Cell Rep (2020) 31(7):107628. doi: 10.1016/j.celrep.2020.107628 32433953 PMC7242909

[68] Calvo F Ege N Grande-Garcia A Hooper S Jenkins RP Chaudhry SI. Mechanotransduction and YAP-dependent matrix remodelling is required for the generation and maintenance of cancer-associated fibroblasts. Nat Cell Biol (2013) 15(6):637–46. doi: 10.1038/ncb2756 PMC3836234 23708000

[69] Hanley CJ Waise S Ellis MJ Lopez MA Pun WY Taylor J. Single-cell analysis reveals prognostic fibroblast subpopulations linked to molecular and immunological subtypes of lung cancer. Nat Commun (2023) 14(1):387. doi: 10.1038/s41467-023-35832-6 36720863 PMC9889778

[70] Nayar S Campos J Smith CG Iannizzotto V Gardner DH Mourcin F. Immunofibroblasts are pivotal drivers of tertiary lymphoid structure formation and local pathology. Proc Natl Acad Sci U S A (2019) 116(27):13490–7. doi: 10.1073/pnas.1905301116 PMC6613169 31213547

[71] Nayar S Pontarini E Campos J Berardicurti O Smith CG Asam S. Immunofibroblasts regulate LTalpha3 expression in tertiary lymphoid structures in a pathway dependent on ICOS/ICOSL interaction. Commun Biol (2022) 5(1):413. doi: 10.1038/s42003-022-03344-6 35508704 PMC9068764

[72] Zhu G Nemoto S Mailloux AW Perez-Villarroel P Nakagawa R Falahat R. Induction of tertiary lymphoid structures with antitumor function by a lymph node-derived stromal cell line. Front Immunol (2018) 9:1609. doi: 10.3389/fimmu.2018.01609 30061886 PMC6054958

[73] Ley K. The second touch hypothesis: T cell activation, homing and polarization. F1000Res. (2014) 3:37. doi: 10.12688/f1000research.3-37.v2 25580220 PMC4038319

[74] Cohen M Giladi A Barboy O Hamon P Li B Zada M. The interaction of CD4(+) helper T cells with dendritic cells shapes the tumor microenvironment and immune checkpoint blockade response. Nat Cancer (2022) 3(3):303–17. doi: 10.1038/s43018-022-00338-5 35241835

