# Supplementary figures and images for: Cancer-associated fibroblasts drive CXCL13 production in activated T cells via TGF-beta
Source: Front Immunol. 2023 Jul 13;14:1221532. doi: 10.3389/fimmu.2023.1221532 (PMC10373066; doi:10.3389/fimmu.2023.1221532)

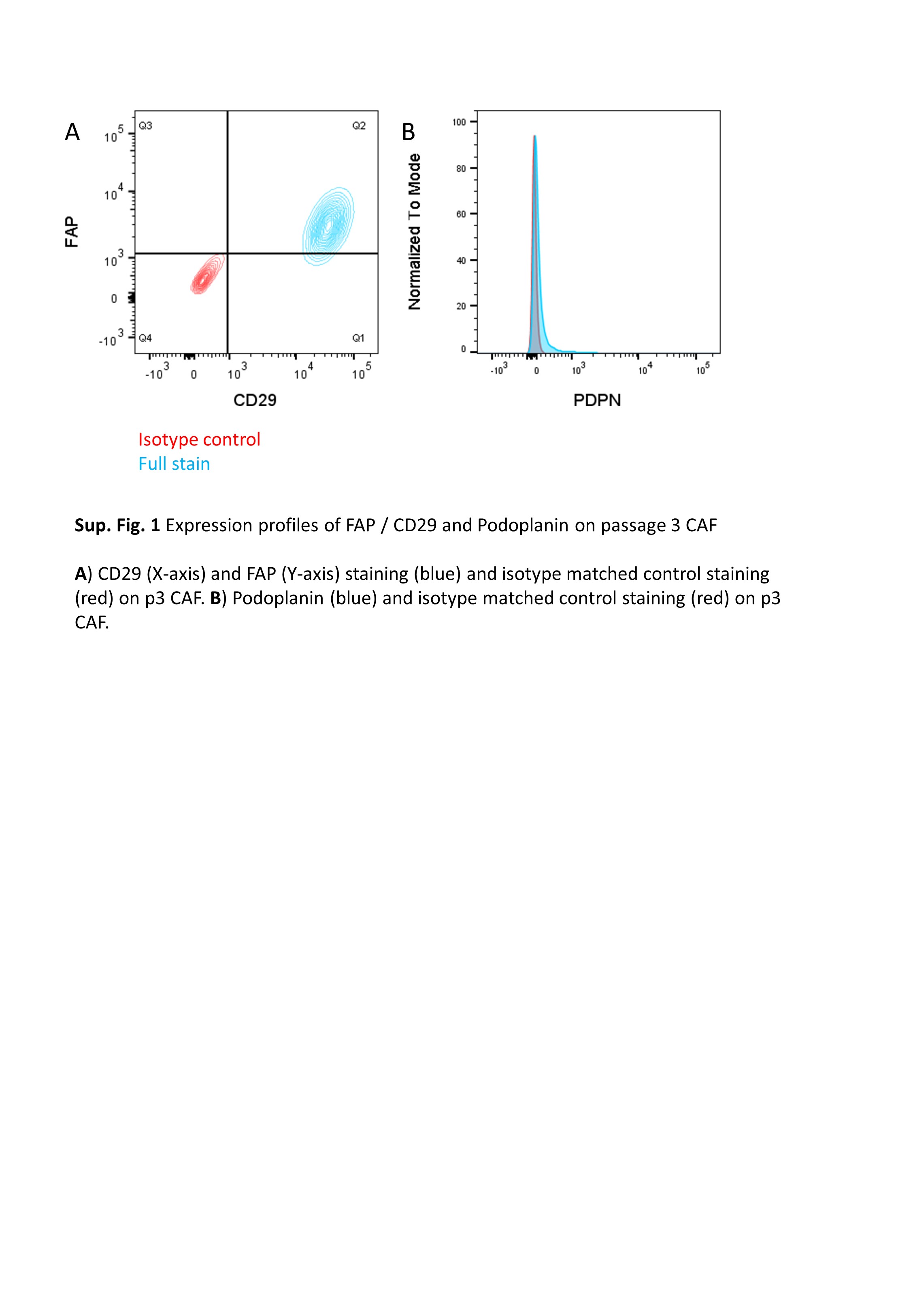

Supplement: Supplementary file 1 [file Image_1.jpeg]

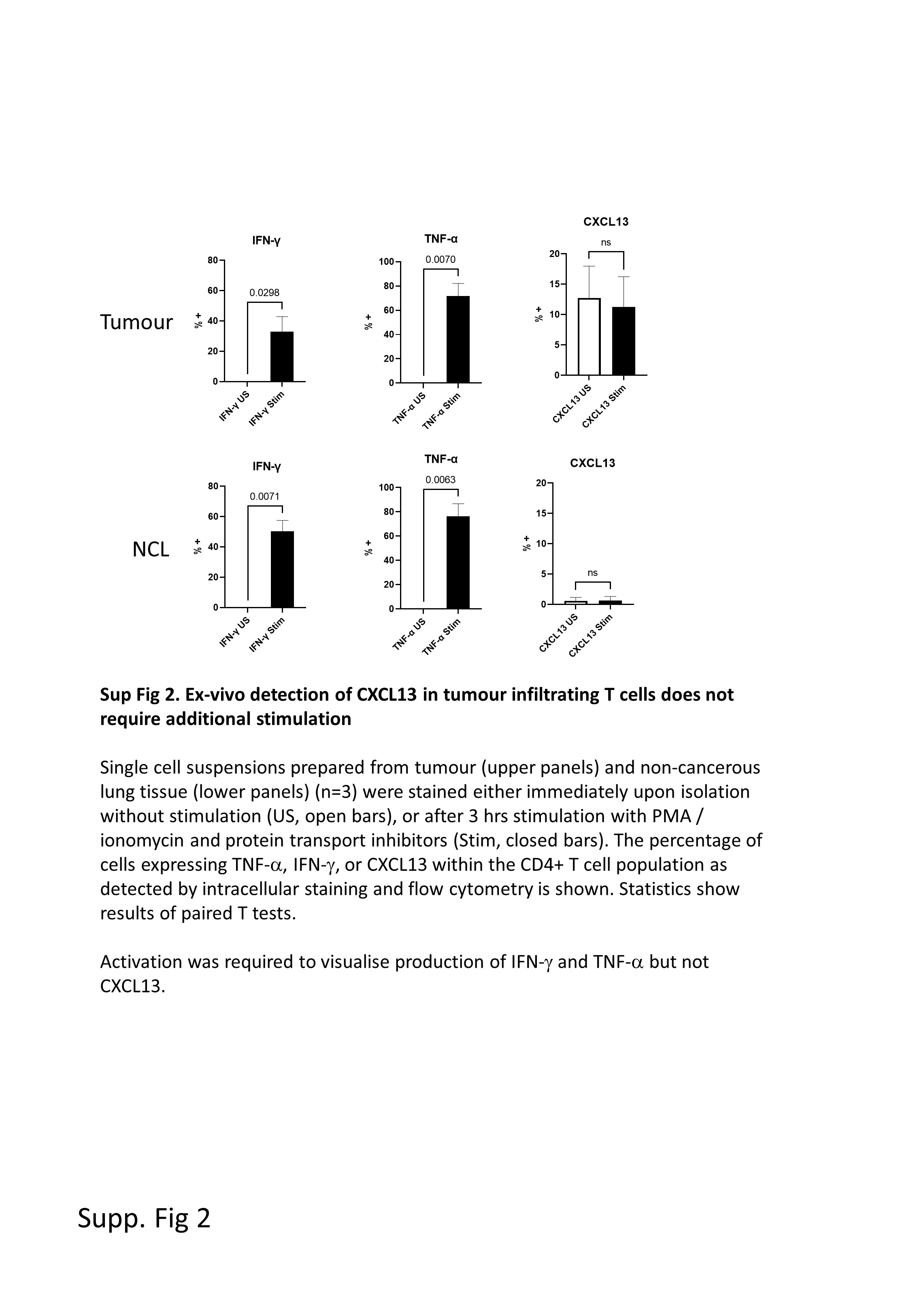

Supplement: Supplementary file 2 [file Image_2.jpeg]

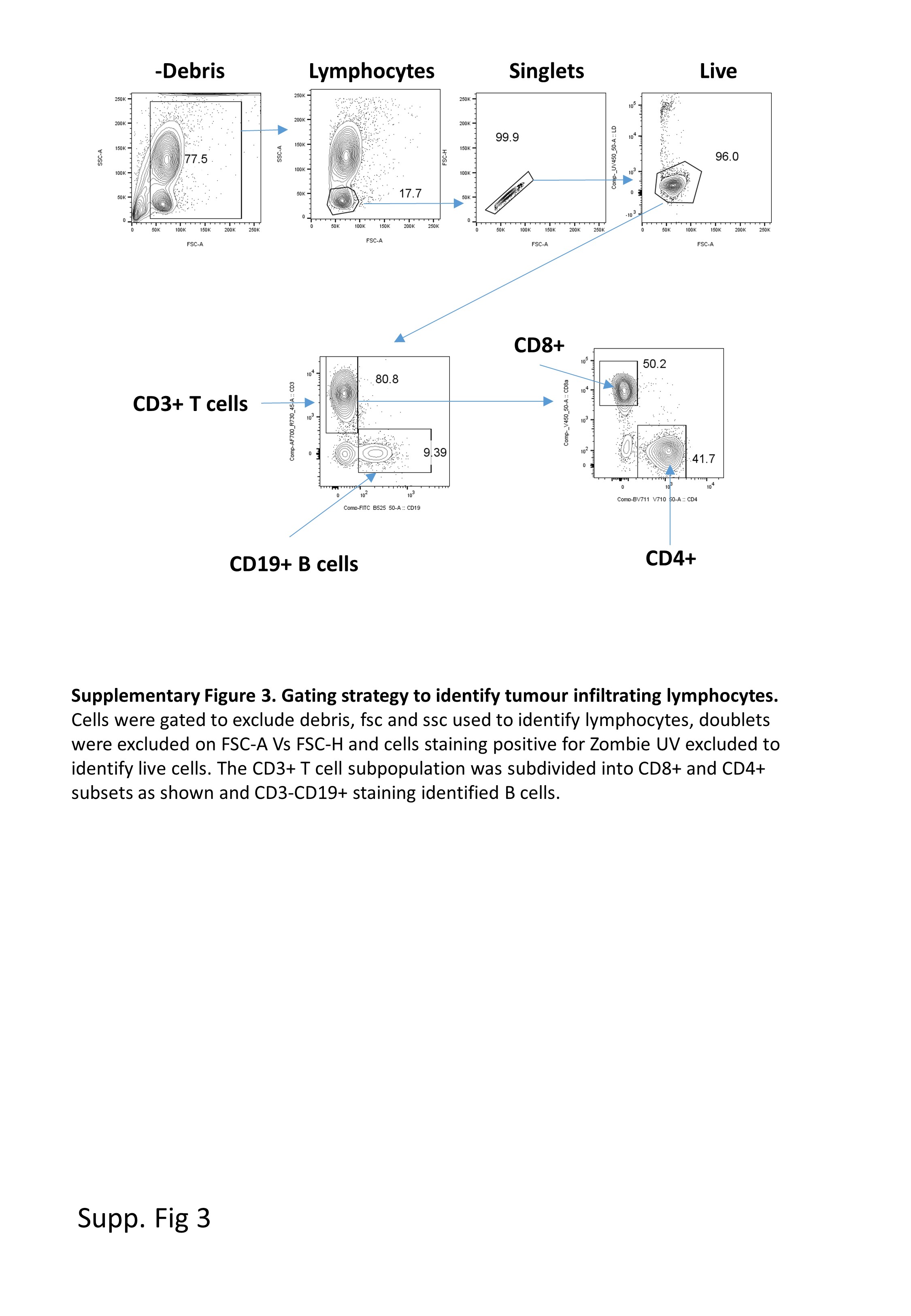

Supplement: Supplementary file 3 [file Image_3.jpeg]

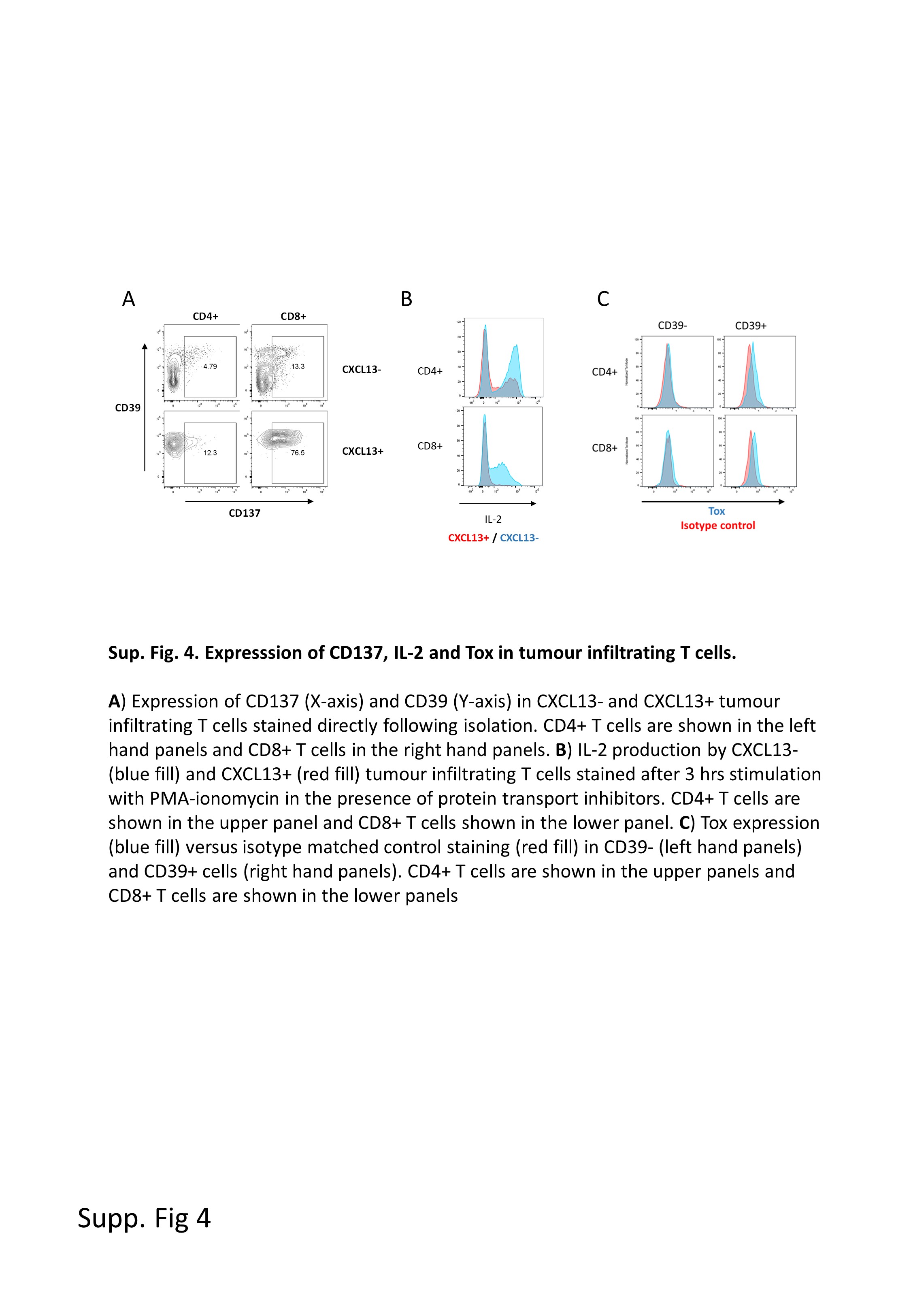

Supplement: Supplementary file 4 [file Image_4.jpeg]
